# Emc3 maintains intestinal homeostasis by preserving secretory lineages

**DOI:** 10.1038/s41385-021-00399-2

**Published:** 2021-03-30

**Authors:** Meina Huang, Li Yang, Ning Jiang, Quanhui Dai, Runsheng Li, Zhaocai Zhou, Bing Zhao, Xinhua Lin

**Affiliations:** 1 State Key Laboratory of Genetic Engineering, School of Life Sciences, Zhongshan Hospital, Fudan University, Shanghai, China; 2 National Health Commission (NHC) Key Laboratory of Reproduction Regulation, Shanghai Institute of Planned Parenthood Research, Fudan University, Shanghai, China

## Abstract

Intestinal exocrine secretory lineages, including goblet cells and Paneth cells, provide vital innate host defense to pathogens. However, how these cells are specified and maintained to ensure intestinal barrier function remains poorly defined. Here we show that endoplasmic reticulum membrane protein complex subunit 3 (Emc3) is essential for differentiation and function of exocrine secretory lineages. Deletion of Emc3 in intestinal epithelium decreases mucus production by goblet cells and Paneth cell population, along with gut microbial dysbiosis, which result in spontaneous inflammation and increased susceptibility to DSS-induced colitis. Moreover, Emc3 deletion impairs stem cell niche function of Paneth cells, thus resulting in intestinal organoid culture failure. Mechanistically, Emc3 deficiency leads to increased endoplasmic reticulum (ER) stress. Mitigating ER stress with tauroursodeoxycholate acid alleviates secretory dysfunction and restores organoid formation. Our study identifies a dominant role of Emc3 in maintaining intestinal mucosal homeostasis.

## Introduction

Intestinal mucosa which consists of physical barrier and luminal immune factors, such as secretory IgA, defensins, lysozyme, Agr2, and Reg3, is the first line of host to defend against external deleterious stimuli.^1^ The physical barrier is composed of a layer of epithelial cells that classified into absorptive and secretory lineages, both of which are constantly replenished by Lgr5+ intestinal stem cells (ISCs). Absorptive enterocytes are the major type of intestinal epithelial cells, making up to 80% of the entire epithelium. The secretory lineages include mucus-producing goblet cells, antimicrobial peptides (AMPs)-secreting Paneth cells, hormone-secreting enteroendocrine cells, and rare infection-mediating tuft cells.^2,3^ Both perturbation of mucosal integrity and reduction in innate immune factors are implicated in the pathogenesis of inflammatory bowel diseases (IBDs).^4–6^

As the most abundant secretory lineage, goblet cells secrete various hydrophilic glycoproteins, including mucins and other protective factors such as Agr2, Zg16, TFF3, FCGBP, and RELMβ, to form a lubricative barrier blocking microbial invasion into intestinal epithelium.^7^ These glycoproteins are synthesized in endoplasmic reticulum (ER), followed by selectively packaged into intercellular granules.^8^ ER plays dominant roles in ensuring proper folding and maturation of mucins, whose disruption accompanies with goblet cell depletion and spontaneous colitis.^9–11^

Paneth cells are intermingled with ISCs at the bottom of crypts in the small intestine. Due to its specialized position, Paneth cells constitute a significant component of the ISC niche by supplying requisite signaling ligands (Wnt, EGF, and Dll4), as well as metabolite (Lactate), for ISC maintenance and differentiation.^12–14^ Paneth cells also serve as a part of the innate immune system by secreting AMPs into gut lumen to influence intestinal microbiome thus to maintain microbiome-host homeostasis.^13,15,16^ It has been reported that elevated ER stress in *Xbp1*^−/−^ Paneth cells leads to Paneth dysfunction and consequent spontaneous enteritis.^17,18^ In addition, several gene mutations that impair Paneth cell function and cause AMPs deficiency, are associated with human Crohn’s disease.^19–22^ Hence, understanding the mechanisms of how goblet cells and Paneth cells maintain their secretory functions would facilitate prevention or treatment of IBDs.

Emc3, encoded by the mouse *Tmem111* gene, is a subunit of the highly conserved ER membrane protein complex (EMC), which is involved in protein folding.^23,24^ Accumulated evidence shows that EMC ensures the biosynthesis, stabilization and/or trafficking of specific multi-pass membrane proteins in *Caenorhabditis* *elegans*, *Drosophila* *melanogaster*, and *Mus musculus*,^25–28^ suggesting that EMC is a component of ER folding machinery critical for protein maturation. A previous study indicates that mammalian Emc3 is required for mouse pulmonary surfactant synthesis and lung function at birth.^27^ However, the function of Emc3 in gut development and homeostasis has not been investigated yet.

In this study, we establish a dominant role of Emc3 in maintaining intestinal secretory lineages and protecting against inflammatory diseases. Emc3-deficient mice are more susceptible to DSS-induced colitis and *Salmonella Typhimurium* infection. Deletion of Emc3 in intestinal epithelium impairs mucus-producing function of goblet cells and differentiation of Paneth cells, which modulate gut bacterial composition. Moreover, Emc3 deletion abolishes intestinal organoid culture through disrupting stem cell niche constructed by Paneth cells. Mitigating ER stress caused by Emc3 deficiency with tauroursodeoxycholate acid (TUDCA) rescues secretory lineages and restores the formation of intestinal organoids.

## Results

### Emc3 deletion leads to spontaneous inflammation and increased susceptibility to induced colitis

**Fig. 1:**
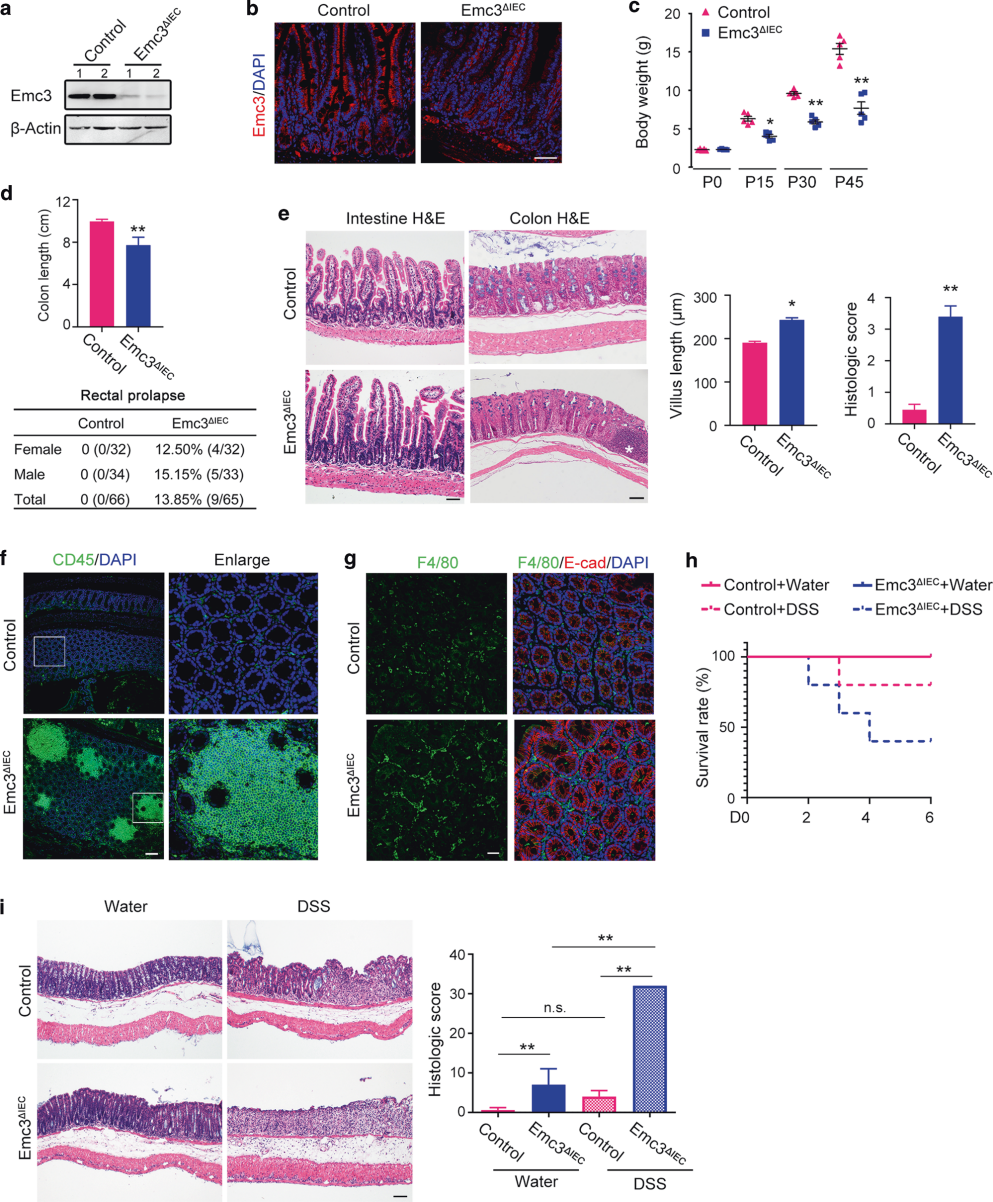
Emc3 deletion leads to spontaneous inflammation and increased susceptibility to DSS-induced colitis. Pattern of Emc3 expression detected by western blot (**a**) and immunofluorescence (**b**) from 8-week-old control and *Emc3*^*ΔIEC*^ ileum. *n* = 2 independent samples for each genotype. β-Actin was used as a loading control. Scale bar, 50 μm. **c** Body weight comparison between control and *Emc3*^*ΔIEC*^ mice at indicated time points. Data represent mean ± SEM. *n* = 5 for each genotype. Wilcoxon’s rank sum test: **p < 0.01. *p < 0.05. **d** Upper panel: length of colon (cm). *n* = 5 for each genotype. Student’s *t*-test: **p < 0.01. Lower panel: the incidence of rectal prolapse determined in male and female mice (8–16 weeks, *n*, indicated). **e** Morphology of intestinal mucosa revealed by H&E staining. Right panel: the length of villus and histologic score of colitis. *n* = 5 for each genotype. Data represent mean ± SEM. Student’s *t*-test: **p < 0.01, *p < 0.05. Scale bar, 50 μm. Asterisk indicates an inflammation focus in mutant colon. Representative images of colonic sections stained with leukocytes (**f**) and macrophages (**g**) marker. E-cad was used to visualize epithelium. Scale bar, 50 μm. **h** Kaplan–Meier survival curve during DSS treatment. **i** Colonic sections stained by H&E and histological scores of DSS-induced colitis. *n* = 3 for per group. Statistical data represent mean ± SEM. One-way ANOVA: **p < 0.01, n.s. not significant. Scale bar, 50 μm.

We first detected the expression of Emc3 in intestinal mucosa using a specific antibody against N terminal of Emc3 protein (Fig. 1a). We found that Emc3 exhibited a ubiquitous expression pattern along the villus-crypt axis in intestinal epithelium (Fig. 1b), while its expression level was much lower in laminar propria.

To investigate the function of Emc3 in gut development and homeostasis, we generated Emc3 floxed mice by inserting two loxp sites into upstream of exon 4 and downstream of exon 5 (Supplementary Fig. 1a),^27^ then employed *Villin-Cre* to generate gut epithelium-specific deletion of *Emc3* (*Emc3*^*fl/fl*^*; Vil-Cre*, henceforth refer to as *Emc3*^*ΔIEC*^). *Emc3*^*ΔIEC*^ mouse pups were born at expected Mendelian frequency and showed no gross phenotype at birth. We validated the efficient depletion of Emc3 at both protein and mRNA level by immunoblots (Fig. 1a) and quantitative PCR (qPCR) (Supplementary Fig. 1b). Immunostaining further verified that Emc3 expression was removed from *Emc3*^*ΔIEC*^ intestinal epithelium, but not from laminar propria or other tissues (Fig. 1b).

Emc3-deficient mice started to display decreased body weight within 2 weeks of age relative to their littermates (Fig. 1c), and such reduction in the growth rate was gradually obvious. *Emc3*^*fl/+*^*; Vil-Cre* mice were used as control, as no defect was observed compared to wild-type mice. *Emc3*^*ΔIEC*^ and control littermates were used for the following study. There was no difference in the length of small intestine between control and mutant mice, suggesting that gastroenterology development was not delayed after Emc3 depletion (Supplementary Fig. 1c). However, the gastrointestinal tract of *Emc3*^*ΔIEC*^ mice exhibited signs of chronic inflammation, including dilated and shortened colon, and rectal prolapse as early as 8 weeks (Fig. 1d). We further investigated the gut histology by hematoxylin and eosin (H&E) staining. *Emc3*^*ΔIEC*^ mice showed normal appearance of intestinal architecture, whereas increased intestinal villus length and a higher histologic score for colonic inflammation were observed in *Emc3*^*ΔIEC*^ mice (Fig. 1e). Meanwhile multifocal aggregates of inflammatory cells (CD45+ leukocytes and F4/80+ macrophages) were appreciated in *Emc3*^*ΔIEC*^ colon (Fig. 1f, g), consistent with spontaneous inflammation observed above.

Next, we evaluated whether Emc3 played a protective role against DSS-induced colitis. Both control and *Emc3*^*ΔIEC*^ mice were conducted with oral DSS administration for 6 days. Following the treatment, mutant mice exhibited remarkable symptoms of colitis including increased mortality, severe weight loss, inflamed colon, and increased intestinal permeability (Fig. 1h, Supplementary Fig. 2a–c). H&E staining of colonic sections showed an extensive loss of crypts in mutant mice, and pathological analysis further confirmed Emc3-deficient mice were more susceptible to DSS-induced colitis (Fig. 1i).

We also examined the effect of Emc3 deletion on defense against enteric pathogen *S. Typhimurium* (S. Tm). After clearance of commensal gut flora by streptomycin, S. Tm enables to colonize mouse intestine, followed by penetrating epithelial barrier through M cells and spreading to liver and spleen.^29^ At 8 weeks of age, control and mutant mice were orally infected with 2 × 10^8^ colony-forming unit (CFU) S. Tm. As expected, *Emc3*^*ΔIEC*^ mice displayed more bacterial burdens in their liver and spleen compared to infected-control mice (Supplementary Fig. 2d). Collectively, we demonstrate that intestinal epithelial Emc3 plays a protective role against colitis and pathogen infection.

### Emc3 deletion impairs mucus production by goblet cells

**Fig. 2:**
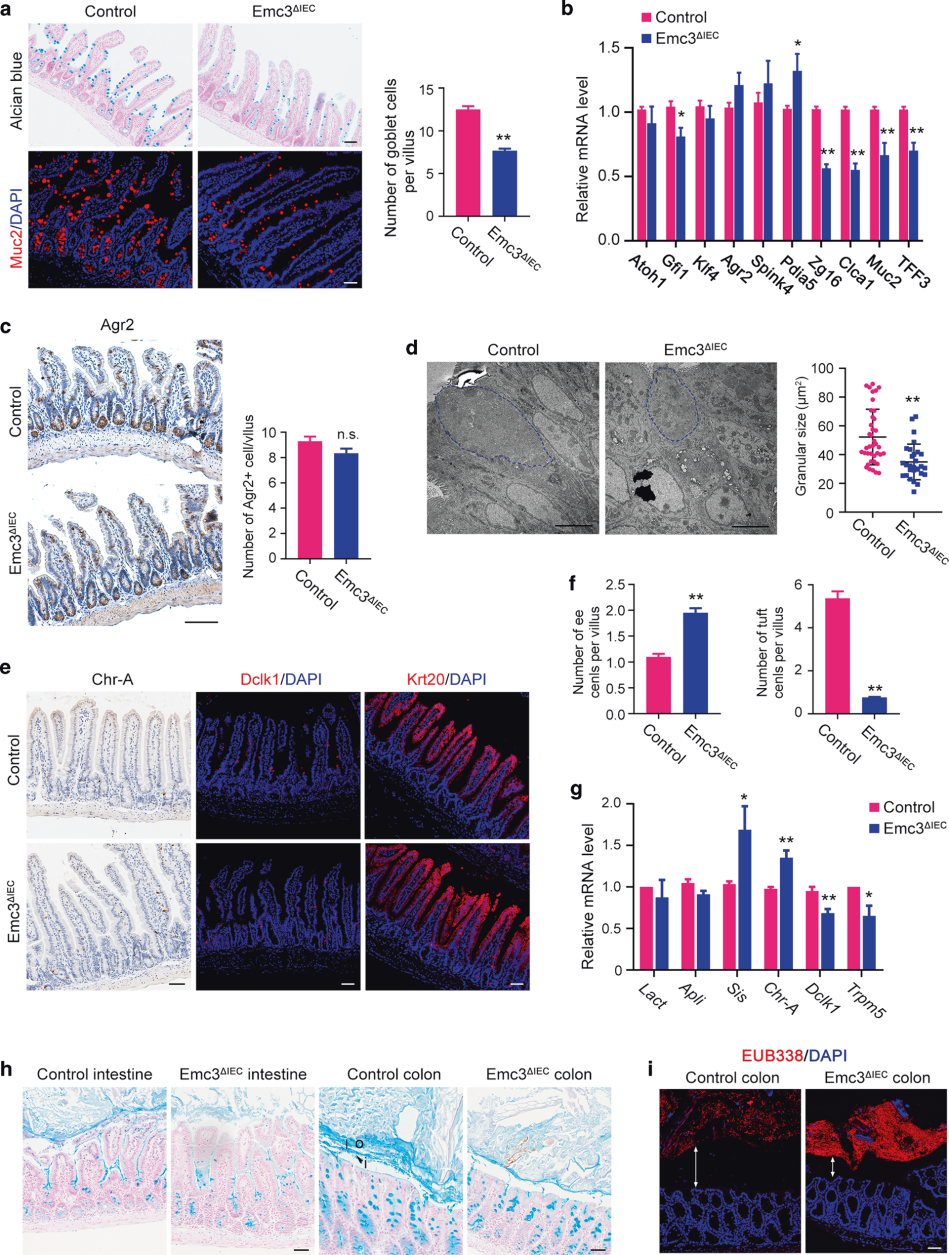
Emc3 deficiency impairs the mucus-producing function of goblet cells. **a** Representative images of goblet cells in ileal sections stained with Alcian blue and Muc2. Scale bar, 50 μm. Right panel: quantification of Muc2+ goblet cells. *n* = 3 for each genotype. **b** Expression of goblet cell markers quantified by qPCR. *n* = 6 for each genotype. **c** Immunohistochemistry and quantification of Agr2+ goblet cells in the ileum of control and *Emc3*^*ΔIEC*^ mice. *n* = 4 for each genotype. Scale bar, 100 μm. **d** Transmission electron microscopy (TEM) images and the measurement of average granular size per goblet cell. Representative of *n* = 3 for each genotype. Scale bar, 5 μm. Secretory granules in goblet cells, blue outline. Representative images (**e**) and quantification (**f**) of immunostained enteroendocrine cells (ee cells), tuft cells, and enterocytes in ileal sections. *n* = 3 for each genotype. Scale bar, 50 μm. **g** Expression of specific markers for enterocytes (*Lact*, *Apli*, *Sis*), enteroendocrine cells (*Chr-A*), and tuft cells (*Dclk1*, *Trpm5*). *n* = 6 for each genotype. **h** Carnoy’s-fixed, paraffin-embedded sections stained with Alcian blue for visualization of mucus layers. Scale bar, 50 μm. o outer mucus layer, i inner layer. **i** The distribution of microbes analyzed by in situ hybridization with Eubacteria-specific (red) probes, counterstained with DAPI. Representative of *n* = 3 for each genotype. Scale bar, 50 μm. Bidirectional arrows indicate the distance between commensal bacteria and host. **a**–**g** Statistical data represent mean ± SEM. Student’s *t*-test: **p < 0.01, *p < 0.05, n.s. no significant.

Considering the secretory lineages of intestinal epithelium highly depend on the function of ER, we speculated that loss of Emc3 would impair the composition of epithelial population. *Emc3*^*ΔIEC*^ villi showed evident reduction in goblet cell density and size as seen by Alcian blue and Muc2 staining (Fig. 2a). The reduction of mucus-producing goblet cells was also observed in mutant colonic sections (Supplementary Fig. 3a). Consistently, qPCR revealed significant downregulation of typical mucus components, including *Clca1*, *Zg16, Muc2*, *TFF3*, after Emc3 depletion. In contrast, other goblet cell marker genes (*Agr2*, *Spink4*, and *Pdia5*) and transcription factors required for goblet cell differentiation (*Klf4*), were comparably expressed (Fig. 2b). Immunostaining and quantification of Arg2 positive goblet cells demonstrated that there was no significant difference in the number of Agr2+ goblet cells between control and *Emc3*^*ΔIEC*^ (Fig. 2c). To better characterize the effect of Emc3 deficiency on goblet cells, we used transmission electron microscopy (TEM). *Emc3*^*ΔIEC*^ mice displayed smaller mucous granules in most goblet cells compared to control (Fig. 2d), indicating defective mucus biogenesis and trafficking. These data suggest that Emc3 deficiency impairs secretory function of goblet cells, while the goblet cell lineage identity is maintained.

We further examined the effects of Emc3 depletion on other cellular lineages. Immunostaining and qPCR demonstrated that Emc3 deficiency resulted in increased enteroendocrine cell population (marked by Chr-A), while tuft cell population was strongly decreased (marked by Dclk1) (Fig. 2e–g). The pan-differentiation of enterocytes (marked by Krt20) was comparable between control and *Emc3*^*ΔIEC*^ (Fig. 2e, g).

Mouse colon contains two mucus layers with distinct properties: a firm inner layer that is sterile and a looser outer layer harboring diverse bacterium.^30^ Given that goblet cells were affected in *Emc3*^*ΔIEC*^ colon, we asked whether the colonic mucus barrier was influenced by Emc3 depletion. Corresponding to comprised mucus producing by goblet cells, mucus layers in ileum and colon were disorganized and thinner in mutant compared to control (Fig. 2h). In addition, fluorescent in situ hybridization (FISH) with a 16S rRNA probe (EUB338) was performed to detect the residence of gut microbiota. The exclusion of bacteria from the inner mucus layer was confirmed both in control and mutant mice (Fig. 2i), but there were more bacteria present in mutant gut lumen relative to control. Our data suggest that ablation of Emc3 impairs secretory cell population in intestinal villi and disrupts the mucus barrier.

### Emc3 is essential for the differentiation and maintenance of Paneth cells

**Fig. 3:**
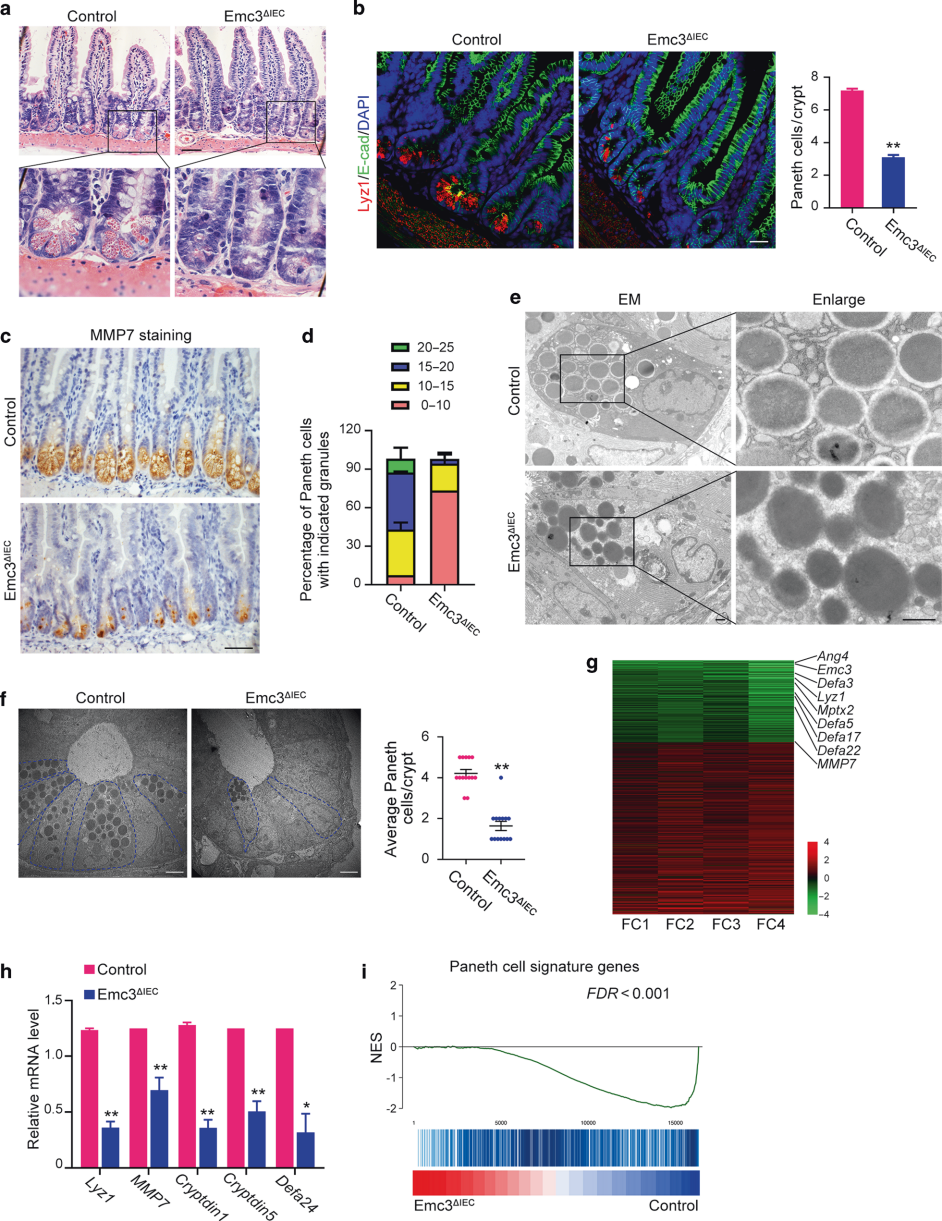
Emc3 is essential for Paneth cell differentiation and function. **a** Representative images of H&E stained ileal sections. Scale bar, 50 μm. **b** Mouse ileum stained with Paneth cells marker (Lyz1) together with E-cad to visualize the cellular boundary. Right panel: quantification of Lyz1+ Paneth cells. *n* = 3 for each genotype. Statistical data represent mean ± SEM. Student’s *t*-test: **p < 0.01. Scale bar, 50 μm. **c** Immunohistochemistry staining for MMP7 on intestinal sections. Scale bar, 50 μm. **d** Paneth cells with distinct granule numbers. *n* = 3 for each genotype. Stacked barplots represent the mean percentage of number ± SEM. **e** Representative TEM images of Paneth cell granules in control and mutant crypts. Scale bar, 1 μm. **f** Representative TEM images of crypt bases. Paneth cells, blue outline. *n* = 3 for each genotype. Student’s *t*-test: **p < 0.01. Scale bar, 5 μm. **g** RNA-seq of intestinal crypts. The top downregulated genes in Emc3-deficient crypts are listed. **h** Paneth cell markers detected by qPCR. The statistical data represent mean ± SEM. *n* = 6 for each genotype. Student’s *t*-test: **p < 0.01, *p < 0.05. **i** Gene set enrichment analysis (GSEA) of Paneth cell signature genes, presented as normalized enrichment score (NES).

We next assessed whether loss of Emc3 affected cells at the crypt base. Examination of H&E stained sections revealed an absence of eosinophilic secretory granules at the base of *Emc3*^*ΔIEC*^ crypts, indicating that Paneth cells were impaired after Emc3 deletion (Fig. 3a). Immunostaining of lysozyme 1 (Lyz1) and matrix metalloproteinase 7 (MMP7) confirmed that Emc3 ablation resulted in loss of Paneth cells (Fig. 3b, c).

Paneth cell secretory granules contain a variety of AMPs, including Lyz1, α-defensins (Defas), angiogenin-4 (Ang4), secretory phospholipase A2 (sPLA2), et al.^31^ Quantification of granule numbers showed that the majority of wild-type Paneth cells had ≥15 granules per cell (55.31% of total Lyz1+ cells in control), while rare mutant Paneth cells had ≥15 granules per cell (3.15% of total Lyz1+ cells in *Emc3*^*ΔIEC*^) (Fig. 3d). TEM was performed to evaluate how secretory function of Paneth cells was influenced. Abundant electron-dense core vesicles (DCVs) with obvious halo surrounding were present in wild-type Paneth cells (Fig. 3e). However, DCVs in mutant sections were reduced both in their number and size, indicative of immature Paneth cells. As murine Paneth cell granules are coated by Muc2,^32^ the presence of immature secretory vesicles might be due to Muc2 reduction in Emc3-deficient mice. Besides, according to its ultrastructural features (electron-dense core granules and prominent perinuclear ER) in TEM, Paneth cell population decreased from 4.21 ± 0.70 cells per crypt in control to 1.64 ± 0.84 cells per crypt in mutant (Fig. 3f). Our data suggest that Emc3 is required for Paneth cell population.

We further analyzed the molecular changes of *Emc3*^*ΔIEC*^ crypts by RNA sequencing. Our data revealed that after Emc3 deletion 472 genes were differentially expressed, among which 172 genes were downregulated (Supplementary Fig. 4a). Of note, abundance of Paneth cell marker genes, represented by *Lyz1*, *MMP7*, and *Defas*, was significant decreased upon Emc3 deletion (heatmap in Fig. 3g, qPCR validation in Fig. 3h). In addition, gene set enrichment analysis (GSEA) further confirmed the downregulation of Paneth cell signature genes in mutant crypts (Fig. 3i). Together, our data indicate that Emc3 is essential for Paneth cell differentiation.

### Emc3 deletion impairs the niche role of Paneth cells to support ISCs

**Fig. 4:**
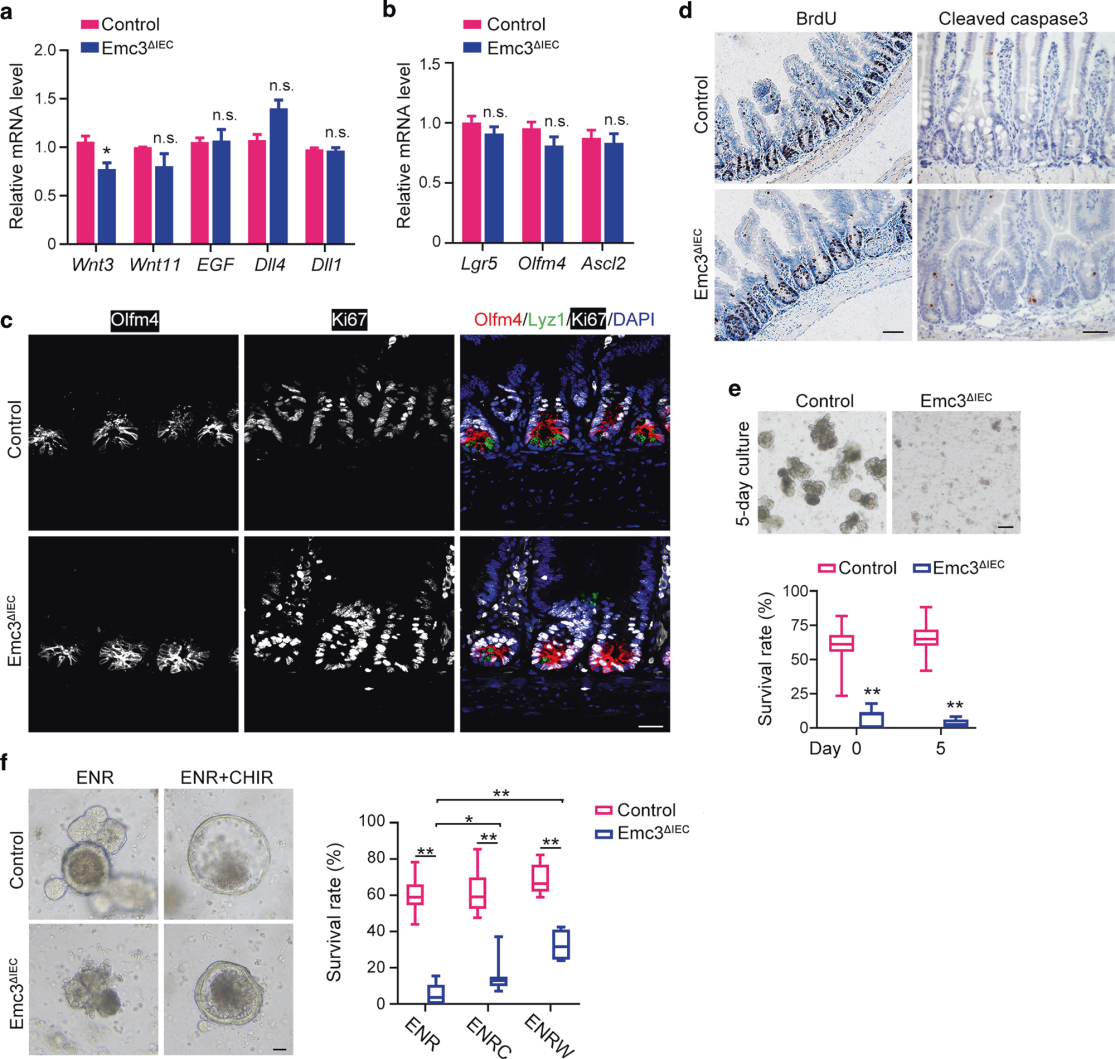
Emc3 deletion ablates the Paneth niche support for intestinal stem cells. Relative expression of growth factors/ligands (**a**) and intestinal stem cell markers (**b**) in isolated epithelial cells from control and *Emc3*^*ΔIEC*^ crypts. *n* = 6 for each genotype. The statistical data represent mean ± SEM. Student’s *t*-test: *p < 0.05, n.s. no significant. **c** Co-immunostaining of ileal sections for proliferative marker (Ki67), stem cell marker (Olfm4), Paneth cell marker (Lyz1). Scale bar, 50 μm. **d** Proliferation and apoptosis detected by BrdU (2 h after injection) and Caspase3 staining, respectively. Scale bar, 50 μm. **e** Representative images of intestinal organoids generated from isolated crypts and cultured for 5 days. Lower panel: quantification of organoid formation ratio. Data are shown as box-and-whisker plots. *n* = 4 for each group. Student’s *t*-test: **p < 0.01. Scale bar, 100 μm. **f** Representative images of intestinal organoids in the presence of 3 μM CHIR99021 or vehicle for 5 days. Right panel: organoid formation ratios. Data are shown as box-and-whisker plots. *n* = 4 for each group. One-way ANOVA: **p < 0.01, *p < 0.05. Scale bar, 10 μm. E EGF, N Noggin, R R-Spondin1, C CHIR99021, W Wnt3A.

Paneth cells constitute niche for ISCs by producing niche factors. As we described above, Emc3 is required for Paneth cell function. Therefore, we asked whether Emc3 depletion caused loss of ISCs/progenitor cells. First, qPCR revealed a profound decrease in the expression of *Wnt3a* (Fig. 4a), without changes in *Wnt11, EGF*, *Dll4*, and *Dll1* expression after Emc3 depletion. Unexpectedly, mRNA levels of ISC marker genes, such as *Lgr5*, *Olfm4*, and *Ascl2* and immunofluorescent staining of Olfm4 were comparable between control and Emc3-decifient crypts (Fig. 4b, c). GSEA further confirmed that stem cell pool in *Emc3*^*ΔIEC*^ was not affected in vivo (Supplementary Fig. 4b).

Next, we investigated if loss of Emc3 affected the proliferation and apoptosis of ISCs. Both Ki67 staining and short-time BrdU incorporation assay revealed no difference in the proliferation of crypt cells (Fig. 4c, d). However, 24 h after intraperitoneal injection, BrdU positive cells migrated higher toward the villus tip in Emc3-deficient mice compared to control, suggesting higher migration capacity after Emc3 depletion (Supplementary Fig. 4c). Moreover, assessed by cleaved Caspase3 staining and TUNEL, *Emc3*^*ΔIEC*^ mice displayed increased apoptotic cells in the crypts (Fig. 4d, Supplementary Fig. 4d).

Paneth cells are demonstrated to be dispensable for stem cell maintenance in vivo, as other alternative cell types providing essential factors to support stem cells.^33,34^ To rule out the influence of mesenchymal cells, we ascertained whether Emc3 deletion impaired functional stem cell activity by organoid culture. When cultured in ENR (EGF, R-Spondin1, and Noggin) medium, crypts from control mice formed organoids and grew in size with extensive budding (Fig. 4e), indicating that stem cells were proliferating and differentiating. However, isolated crypts from *Emc3*^*ΔIEC*^ mice failed to generate organoids and underwent atrophy, exhibiting reduced organoid-forming capacity.

In our above data, Wnt3a expression was decreased after Emc3 deletion. Therefore, we hypothesized that activation of Wnt signaling might restore the organoid formation from mutant crypts. To this end, we treated organoids from control and mutant mice with CHIR99021, a GSK-3β inhibitor, to stimulate Wnt signaling. As expected, control organoids with higher Wnt signaling grew bigger and formed less buds as previously reported (Fig. 4f).^35^ Strikingly, CHIR administration, as well as recombinant Wnt3A, rescued the organoid-forming capacity of Emc3-deficient crypts, supporting the paradigm that decreased Wnt support for ISCs leads to organoid culture failure ex vivo.

### Elevated ER stress and UPR activation caused by Emc3 deficiency disrupts secretory lineages

**Fig. 5:**
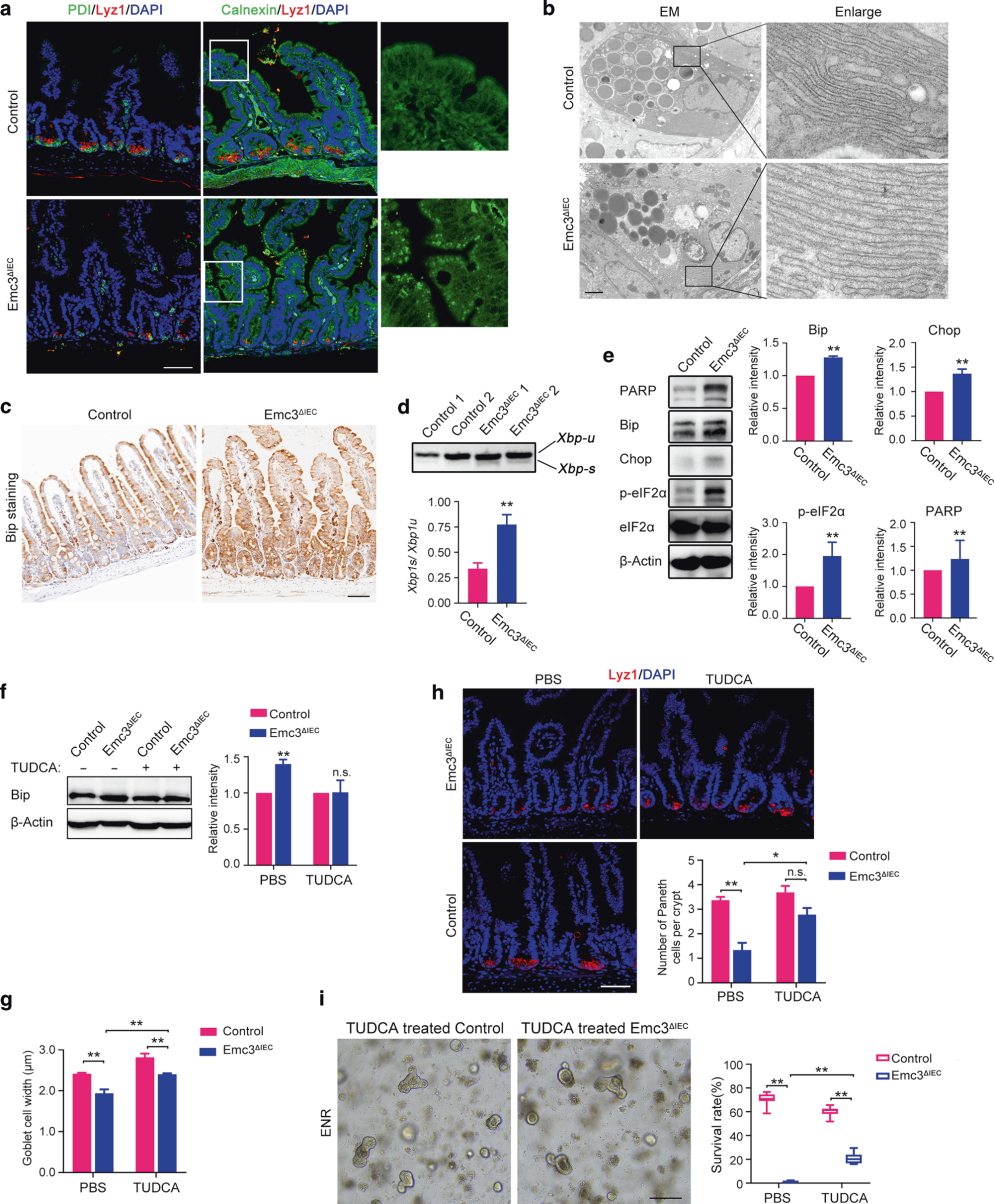
Elevated ER stress and UPR activation caused by Emc3 deficiency disrupts secretory cells. **a** Representative co-immunofluorescent images for Paneth cell marker (Lyz1) and ER markers (PDI or Calnexin) on ileal sections. Enlarged images of Calnexin in the villus are shown. Scale bar, 50 μm. **b** Representative TEM images of ER in Paneth cells. Note distend ER after Emc3 depletion. Scale bar, 2 μm. **c** Immunohistochemistry analysis for Bip. Scale bar, 50 μm. **d** Representative PCR results for *Xbp1* splicing in control and mutant crypts. *Xbp1-u*, unspliced form; *Xbp1-s*, spliced form. The upper panel shows two independent mice for each genotype. Lower panel: *n* = 3 for each genotype. **e** Immunoblotting with indicated UPR mediators. β-Actin was used as a loading control. eIF2α was used as a loading control for phospo-eIF2α. *n* = 3 for each genotype. **f** Protein lysate from TUDCA-treated control and mutant crypts immunoblotted with Bip antibody. β-Actin was used as a loading control. *n* = 3 for each group. **g** The average width of the goblet cells after TUDCA treatment. *n* = 3 for each group. **h** Immunostaining and quantification of Paneth cells after TUDCA treatment. *n* = 3 for each group. Scale bar, 50 μm. **i** Representative and graphs for organoids generated from TUDCA or PBS administrated mice cultured for 3 days. *n* = 3 for each group. Scale bar, 200 μm. Data are shown as mean ± SEM (**d**–**h**) and box-and-whisker plots (**i**). Student’s *t*-test (**d**–**f**) and One-way ANOVA (**g**–**i**): **p < 0.01, *p < 0.05, n.s. not significant.

Emc3 is one subunit of conserved EMC complex. Therefore, we investigated the effect of Emc3 deletion on the function of ER in intestinal epithelium. Immunostaining for ER marker protein disulfide-isomerase (PDI) showed a strong staining pattern in Lyz1+ Paneth cells in control (Fig. 5a, left panel), consistent with intensive secretory function of Paneth cells. However, Emc3 deletion abolished PDI staining at the bottom of crypts. Another ER marker Calnexin was concentrated in small puncta in the cytoplasm of *Emc3*^*ΔIEC*^ epithelial cells (Fig. 5a, right panel), in contrast to widespread cytoplasmic distribution in control. Our data suggest that ablation of Emc3 leads to disorganization of ER in intestinal epithelium.

TEM was applied to further validate the defect of ER structure in *Emc3*^*ΔIEC*^ crypts. We observed that ER in mutant Paneth cells became more dilated compared to that in control (Fig. 5b), indicating accumulation of proteins in ER after Emc3 depletion. Misfolded protein resident in ER would cause ER stress and induce unfolded protein response (UPR). Indeed, we observed an accumulation of Bip, an ER stress component, in Emc3-deficient epithelial cells (Fig. 5c). Meanwhile, elevated *Xbp1* mRNA splicing (*Xbp1-s*) (Fig. 5d), and increased protein levels of Chop, phospho-eIF2α, and PARP in Emc3-diecient epithelium indicated the activation of UPR signaling (Fig. 5e).

In a recent study, induced ER stress by Xbp1 deletion could cause significant Paneth cell and goblet cell loss,^17^ reminiscent of the hypoplasia of Paneth cells and goblet cells in *Emc3*^*ΔIEC*^ mice. To figure out whether ER stress contributed to the secretory lineage defects, we treated mice with ER stress inhibitor TUDCA.^36^ 3-week-old *Emc3*^*ΔIEC*^ and control littermates were administered 500 mg/kg/day TUDCA by intraperitoneal injection for 6 days. After administration of TUDCA, Bip was significantly downregulated (Fig. 5f, Supplementary Fig. 5a). Mucus production by goblet cells was elevated in TUDCA-treated Emc3-deficient villus compared to vehicle-treated villus (Fig. 5g). Importantly, the number of Lyz1+ Paneth cells was significantly recovered in mutant (Fig. 5h).

Since Paneth cells were restored by inhibition of ER stress in *Emc3*^*ΔIEC*^ mice, we speculated that TUDCA could promote intestinal organoid formation from Emc3-deficient crypts. As expected, TUDCA administration efficiently rescued *Wnt3* expression (Supplementary Fig. 5b). Consistently, 22.5% crypts from TUDCA-treated *Emc3*^*ΔIEC*^ mice survived for 3 days of culture compared to 0.8% crypts from vehicle-treated *Emc3*^*ΔIEC*^ mice (Fig. 5i). These data indicate that releasing ER stress induced by Emc3 depletion restore the differentiation and function of secretory lineages.

### Emc3 depletion causes microbiota dysbiosis

**Fig. 6:**
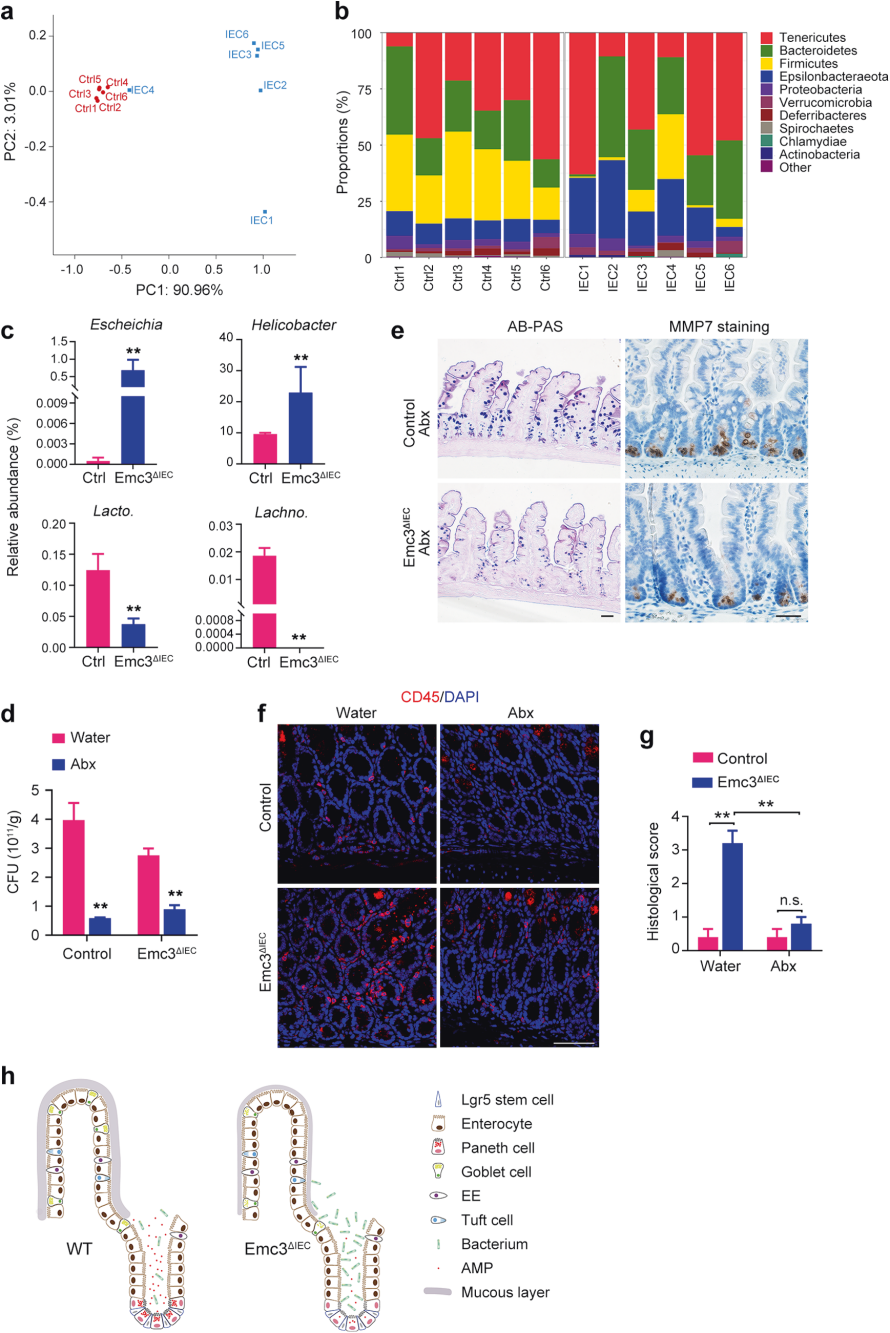
Emc3 maintains the gut barrier function to prevent pathogen infection. **a** Principal coordinate analysis of gut microbial community. **b** Composition of cecal microbiota at phylum level between *Emc3*^*ΔIEC*^ and control littermates. **c** Relative abundance of *Escheichia*, *Helicobacter*, *Lactobacillus*, and *Lachnospiraceae*. Statistical data represent mean ± SEM. Student’s *t*-test: **p < 0.01. **d** Absolute bacterial loads in the feces from *Ad libitum* and Abx fed control and mutant mice. Statistical data represent mean ± SEM. Student’s *t*-test: **p < 0.01. **e** Representative images of goblet cells (AB-PAS) and Paneth cells (MMP7) from mice treated with antibiotics. Scale bar, 50 μm. **f** CD45 staining of colonic sections from water and Abx treated *Emc3*^*ΔIEC*^ and control mice. Scale bar, 50 μm. **g** Histological scores of water and Abx treated colon. *n* = 3 for each group. Statistical data represent mean ± SEM. One-way ANOVA: **p < 0.01, n.s. no significant. **h** Model depicting the role of Emc3 in intestinal homeostasis. In wild-type intestinal epithelium, Emc3 is required for proper epithelial cell differentiation and function, especially for goblet cells and Paneth cells. Emc3-deficient epithelium is devoid of Paneth cells and has decreased mucus production by goblet cells, resulting in gut dysbiosis and being susceptible to inflammation.

AMPs produced by Paneth cells and mucins secreted by goblet cells are implicated in shaping the gut microbiota. Therefore, we detected the composition of the microbiota from control and mutant mice by 16S rRNA-based microbial profiling analysis. *Emc3*^*ΔIEC*^ and control littermates (*n* = 6 for each genotype) were separated according to genotype after weaning and housed until 10 weeks of age. Mice with the same genotype were randomly separated in 2 cages (3 mice in 1 cage) to avoid “cage effect”. Cecal contents were collected and bacterial 16S rRNA amplicons were profiled. A principal coordinate analysis revealed that overall microbial composition of *Emc3*^*ΔIEC*^ mice deviated from that of control group (Fig. 6a). Emc3 deletion led to significant alteration of gut microbiome (Fig. 6b). At the genus level, several potential pathogenic species were more abundant in *Emc3*^*ΔIEC*^, including *Escherichia* and *Helicobacter*. Meanwhile, beneficial bacterial species, such as *Lactobacillus and Lachnospiraceae*, exhibited less abundant in mutant (Fig. 6c). Similarly, ileal microbial composition was obviously changed after Emc3 depletion (Supplementary Fig. 6).

Interactions between host and gut bacteria might be involved in epithelial cell differentiation.^37^ Therefore, we investigated whether secretory lineage defects in Emc3-deficient mice were dependent on changes in microbiota. Treating mice with a cocktail of broad-spectrum antibiotics (Abx) eliminated bacteria in both control and mutant mice (Fig. 6d). The loss of Paneth cells and decreased mucus production were persistent in Abx treated *Emc3*^*ΔIEC*^ mice (Fig. 6e). However, spontaneous inflammation in *Emc3*^*ΔIEC*^ colon was remarkably reduced after antibiotics treatment, illustrating the association of colitis with bacteria dysbiosis after Emc3 depletion (Fig. 6f, g).

## Discussion

Intestinal secretory lineages are responsible for production of mucins and AMPs constituting the first line to defend the invasion of luminal pathogens. Disruption of mucosal homeostasis is always associated with IBDs. Here we show that ablation of Emc3 affects goblet cells in the villus and Paneth cells in the crypt accompanied with elevated ER stress, altogether resulting in attenuated mucosal protective function (Fig. 6h).

Epithelium from murine small intestine and colon share some similarities in their immune functions against vast microbiota. On one hand, mucus system in small intestine and colon modulate host-commensal symbiosis. On the other hand, even though colonic epithelium contains no Paneth cell lineage, it has been reported that Paneth-derived lysozyme immunoactivity could be detected in colonic lumen.^38^ Several mouse genetic studies also demonstrate that Paneth cell depletion or defects in AMPs secretion cause increased susceptibility to DSS-induced colitis.^17,18,39,40^ Therefore, dysfunction of goblet cells and ablation of Paneth cells contribute to altered gut microbiome and colonic inflammation in *Emc3*^*ΔIEC*^ mice.

To date, transcription factors and signaling pathways modulating the specification and maturation of secretory lineages are incompletely defined. Notch signaling is demonstrated to control intestinal secretory lineages.^41,42^ Inhibition of Notch signaling elevates the number of goblet cells.^43^ Besides, the mammalian Target of Rapamycin (mTOR) Complex signaling has been recently shown to regulate the differentiation of intestinal cells in vitro and in vivo.^44,45^ Upregulation of mTOR causes compromised goblet cell and Paneth cell differentiation via elevating NICD and Hes1 level. Here we show that Emc3 deficiency leads to diminished secretory lineages. However, Emc3 depletion has no influence on Hes1 expression examined by qPCR (Supplementary Fig. 3b). Consistently, Math1/Atoh1 is a master regulator of secretory progenitors and is also an antagonist of Hes1 during cell fate decision,^34,46^ whose expression is not altered after Emc3 depletion (Supplementary Fig. 3b), suggesting no changes in the secretory precursor. The zinc-finger transcription factor Krüppel-like factor 4 (Klf4) that regulates epithelial cellular differentiation and promotes terminal differentiation of goblet cells^47,48^ is not affected by Emc3 depletion. Therefore, Emc3 is dispensable for goblet cell differentiation but is required for mucus-producing function of goblet cells.

Unlike goblet cells in the villus, the number of Paneth cells is reduced after Emc3 depletion. Given the evident downregulation of Paneth cell terminal differentiation markers Lyz1 and Cryptdins, as well as reduced size of secretory vesicles of Paneth cells in Emc3-deficient mice, we speculate that Emc3 is implicated in Paneth cell differentiation and/or maturation. It is possible that Emc3-mediated cargo producing function might contribute to Paneth cell terminal differentiation. To support this, increased apoptosis in Emc3-deficient crypts, associated with newly appearing Ki67+ cells at the crypt bottom, suggests that the undifferentiated Paneth cells might be removed through apoptotic clearance. Wnt pathway is implicated in Paneth cell differentiation program,^43,49^ but the relationship between Wnt pathway and Paneth cell differentiation is complex and still not fully elucidated. Sox9 is a target of Wnt signaling and is required for Paneth cell differentiation in intestinal epithelium.^50^ However, expression of Sox9 in *Emc3*^*ΔIEC*^ crypt is not changed compared to control (Supplementary Fig. 3b).

ER stress and UPR signaling are not only the results of abundant protein synthesis, but also may play a role in secretory lineage differentiation. Previous immunohistochemistry staining has shown that the level of ER stress in Paneth cells is heterogeneous, with a subset of Paneth cells expressing high levels of Bip, Xbp1, and phospho-eIF2a,^51^ indicating that ER-UPR signaling potentially regulates a specific stage of Paneth cell differentiation and activity, like in muscle, stomach and immune system.^52^ Notably, ER stress transducer CREB4 acts downstream of Spdef, and plays a role in mediating the differentiation and maturation of goblet cells and Paneth cells.^53^ Similarly, another ER stress transducer OASIS is also reported to promote the differentiation of early goblet cells to mature goblet cells.^54^ Here we show that elevated ER stress blocks Paneth cells differentiation, and suggest that Emc3 is involved in modulation of ER stress to promote the terminal differentiation of Paneth cells, downstream or independent of Wnt-Sox9 axis.

ISCs, residing at the crypt “stem cell niche”, are responsible for generation of the cellular compartment in intestinal epithelium. The expression of stem cell niche factors is not affected in crypt epithelial cells after Emc3 depletion, except for Wnt3a. We speculate that the expression of EGF, Dll4, and Dll1 might be compensated by alternative epithelial cells following loss of Paneth cells. It has been shown that enteroendocrine cells and tuft cells occupy the original Paneth cell position after Paneth elimination and express Notch ligands.^55^ In agreement with unchanged Notch signaling in Emc3-deficient mice, there is no difference in the expression of Olfm4, which is a marker of fast-cycling stem cells/progenitor cells depending on Notch signaling. On the other hand, our data provide the evidence that Paneth cells might be the major epithelial source of Wnts. Consistently, the isolated crypts from *Emc3*^*ΔIEC*^ mice are not able to survive ex vivo, whereas addition of Wnt activators dramatically increases the formation of intestinal organoids from knockout crypt. Together, our study shows that Emc3 is required for intestinal homeostasis and has a protective role against inflammation and bacterial infection.

## Methods

### Mice

Emc3 ^tm1a(EUCOMM)Wtsi^ embryonic stem (ES) cell line carrying a L1L2_Bact_P cassette insertion in Emc3 genomic locus, was purchased from EUCOMM and used to generate mutant mice. Targeted ES cell line was injected into C3H/HeNCrl blastocysts to generate chimeric mice. Germline transmission was verified by mating chimeras with C57Bl6/J (Jackson Laboratories), then progenies were crossed with *Rosa26-flppase* (Stock No: 007844) mice to obtain *Emc3*^*fl/+*^. Mice carrying the Emc3 flox allele were available for Cre-mediated recombination and were backcrossed to obtain homozygous *Emc3*^*fl/fl*^. *Emc3*^*fl/fl*^ mice were bred with *Vil1-Cre* strain (Stock No: 021504) to generate Emc3 mutant mice (*Emc3*^*fl/fl*^; *Vil-Cre*).

Emc3^*ΔIEC*^ mice and littermate controls at 8–16 weeks of age were used to perform the experiments, except for TUDCA and antibiotics experiment. For TUDCA administration, gender-matched 3-week-old mice (*n* = 3 for each genotype in each treatment group) were subjected to TUDCA or vehicle administration, twice daily (250 mg/kg for 8 a.m. and 8 p.m., total 500 mg/kg/day) for consecutive 6 days.

Depletion of commensal bacteria was achieved by feeding 3-week-old mutant mice and their littermates with antibiotic water containing ampicillin (1 mg/ml), neomycin (1 mg/ml), metronidazole (1 mg/ml), and vancomycin (0.5 mg/ml) till 8 weeks (*n* = 3 for each genotype in each treatment group).

All mice were housed in the specific pathogen-free animal care facility at thermal neutral temperature under a 12 h/12 h light/dark cycle. All animal work was performed in accordance to protocols approved by the Fudan University Animal committee.

### DSS-induced colitis and histological analysis

Male mice (*n* = 7 for each genotype) at 8 weeks of age of both control and mutant genotypes were treated for 6 days with drinking water supplemented with 2.5% Dextran Sulfate Sodium (mol. wt. 36,000–50,000 Da, MP Biomedicals). At end of the treatment, mice were sacrificed for histologic analysis.

Body weight loss was calculated as percentage ratio of the original weight at day 0. Colon histology was scored based on 3 parameters as previously described.^56^ Briefly, severity of inflammation (0–3): 0, rare inflammatory cells in the lamina propria; 1, increased numbers of granulocytes in the lamina propria; 2, confluence of inflammatory cells extending into the submucosa; 3, transmural extension of the inflammatory infiltrate. Depth of ulcerations (0–3): 0, absence of ulcer; 1, 1, or 2 foci of ulcerations; 2, 3, or 4 foci of ulcerations; 3, confluent or extensive ulceration. Crypt damage (0–3): 0, intact crypts; 1, loss of the basal one-third; 2, loss of the basal two-thirds; 3, entire crypt loss. Then total scores were multiplied by a factor representing the percentage of tissue involvement: ×1 (0–25%), ×2 (26–50%), ×3 (51–75%), ×4 (76–100%).

### *Salmonella Typhimurium* infection

*S. Typhimurium* (S. Tm) infection was performed as described previously.^57^ Briefly, 8 or 10-week-old mice were fasted for 4 h followed by gastric gavage with 20 mg streptomycin sulfate per animal (*n* = 3 for each genotype). Twenty hours later, mice were fasted for 4 h prior to gastric gavage with 2 × 10^8^ CFU S. Tm (14028 s). Forty-eight hours after infection, spleen and liver were homogenized in PBS with 0.1% Triton X-100 to release intracellular bacteria, and plated on Luria-Bertani (LB) agar after serial dilutions. LB plates were incubated at 37 °C for 24 h before CFU quantification.

### Immunostaining

Small intestine and colonic tissues were fixed in 4% paraformaldehyde in PBS overnight, then embedded in paraffin. For visualization of mucus layer, tissues were fixed in Carnoy’s solution (60% methanol, 30% chloroform, 10% acetic acid) for 4 h and embedded in paraffin. Gut sections of 5 μm were dewaxing and rehydration, then subjected to antigen retrieval in sodium citrate buffer. After blocked in PBST (0.1% Triton-X100) with 5% normal goat serum for 1 h, sections were stained with anti-Emc3 (Sigma-Aldrich, 1:100), anti-Villin (BD Bioscience, 1:500), anti-CD45 (BD Bioscience, 1:100), anti-F4/80 (Bio-rad, 1:200), anti-Muc2 (Santa Cruz, 1:200), anti-Agr2 (Novus, 1:200), anti-Krt20 (CST-13063, 1:300), anti-Chr-A (Santa Cruz, 1:200), anti-Dclk1(Abcam, 1:100), anti-Lyz1 (DAKO, 1:500), anti-MMP7 (Cell Signaling, 1:200), anti-Olfm4 (Cell Signaling, 1:500), anti-PDI (Cell Signaling, 1:200), anti-Calnexin (Cell Signaling, 1:200), anti-Bip (Cell Signaling, 1:200), anti-Ki67 (Abcam, 1:500), anti-BrdU (Millipore, 1:500) and anti-active-Caspase3 (Cell Signaling, 1:200) at 4 °C overnight. For immunofluorescence, sections were stained with Alexa Fluor 488 (JacksonImmunoResearch, 1:400) and Cy3 (JacksonImmunoResearch, 1:400)-labeled secondary antibodies, counterstained with DAPI. Specimens were imaged with Leica LSM 710 or Olympus FV3000 Confocal Laser Scanning Microscope. For immunohistochemistry, sections were incubated with biotinylated secondary antibodies (JacksonImmunoResearch, 1:200). The Vectastain Elite ABC immunoperoxidase detection kit (Vector Labs) was used followed by DAB+ Substrate (Vector Labs) for visualization. Images were obtained using a digital microscope (Leica, Z2 imager).

### Fluorescence in situ hybridization

FISH for eubacterial 16S RNA was performed as previously described.^58^ Briefly, 5 μm Carnoy’s-fixed colonic sections were cut, dewaxing, rehydration and incubated with 5 μg/ml Alexa 546-conjugated EUB338 (5′-*GCTGCCTCCCGTAGGAGT*-3′, Genewiz) in hybridization buffer (0.1 M Tris-HCl, 0.9 M NaCl, 0.1% SDS and 40% formamide, pH 7.2) at 40 °C overnight. The sections were rinsed in washing buffer (20 mM Tris-HCl, 0.9 M NaCl, pH 7.4) and counterstained with DAPI. Images were obtained with Olympus FV3000 Confocal Laser Scanning Microscope.

### In vivo intestinal permeability assay

Mice were fasted for 4 h followed by gastric gavage with 60 mg/100 g body weight of FITC-labeled dextran (4 kD, Sigma-Aldrich). 4 h later, serum was collected and fluorescence intensity (excited at 485 nm and read at 530 nm) was measured by the spectrometer (Biotek synergy^TM^4).

### Crypt isolation and organoid culture

Fresh proximal intestine was dissected, and crypts were isolated as previously described.^59^ Briefly, intestine was opened longitudinally in pre-cold 1 × PBS. After removal of villi by scraping, tissues were minced and incubated in 5 mM EDTA for 30 min at 4 °C. The mixture was agitated and passed through 70 μm cell strainer. Isolated crypts were centrifugated at 600 rpm for 3 min and resuspended in Matrigel (Corning), followed by being plated on a 48 well dish. Standard grow medium Advanced DMEM/F12 (Invitrogen) supplemented with N2 supplement (Invitrogen), B27 supplement (Invitrogen), 1 mM N-Acetylcysteine (Sigma), Glutamax (Invitrogen), Penicilllin-Streptomycin (Invitrogen) and growth factors: 50 ng/ml EGF (R&D Systems), 100 ng/ml Noggin (R&D Systems) and 500 ng/ml R-Spondin1 (R&D Systems). For chemical rescue experiments, 3 μM CHIR99021 (Sigma) or 100 ng/ml recombinant Wnt3A (R&D Systems) was added into the standard medium. Organoid formation analysis was counted manually using bright field microscopy, and presented as the percentage of the number of organoids (day 5) relative to the number of plated crypts (day 0).

### RNA purification and RT-qPCR

Crypt cells were collected and stored in TRIzol (Invitrogen), and total RNA was extracted using Micro RNA extraction kit (Tiangen Biotech) according to the manufacturer’s instructions. For reverse transcription, 1 μg total RNA was used and cDNA was synthesized using GoScript Reverse Transcription System (Promega). qPCR was performed using SYBR Green PCR Master Mix (Bimake) and detected by CFX384 Touch System (Bio-rad). Relative amounts of mRNA were calculated by the comparative Δ*C*_t_ method with *Gapdh* as an internal control. Representative results are shown in the figures. Primer sequences are shown in Supplementary Table 1.

### Immunoblotting analysis

Western blot analysis was carried out on total protein extracts. Samples were homogenized in RIPA cell lysis buffer (Beyotime) supplemented with 1 mM PMSF and protease inhibitor cocktails (Bimake). Tissue lysate was centrifuged at 12,000 rpm for 10 min at 4 °C. Protein supernatant was collected, and the concentration was determined using Rapid Gold BCA Protein Assay Kit (Thermo Scientific). Total 40 μg protein was resolved on 10% polyacrylamide gel and transferred to polyvinylidene fluoride membrane. The membrane was blocked with 5% skim milk in TBST (0.05% Tween20) for 1 h at room temperature, and then incubated with anti-PERK (Cell signaling), anti-Bip (Cell signaling), anti-elF2α (Cell signaling), anti-phospo-elF2α (Cell signaling), anti-PARP (Cell signaling) and anti-β-Actin (Cell signaling) at 4 °C overnight. The membrane was washed three times in TBST and followed by blotting with horseradish peroxidase-conjugated secondary antibody. Protein expression was visualized with the Pierce ECL Western Blotting Substrate (Thermo Scientific) and imaged by ChemiDoc Imaging System (Bio-Rad). The experiments were repeated for at least three times, and representative data were shown. The band intensity was quantitated with BandScan 5.0.

### Transmission electron microscopy (TEM)

Small intestine tissues were collected and fixed overnight at 4 °C in 2.5% glutaraldehyde in 0.1 M PB, and then post-fixed in 1% buffered osmium tetroxide for 2 h. Specimens were processed via a routine procedure and examined under a transmission electron microscope (Hitachi, HT7700).

### *Xbp1* splicing assay

*Xbp1* splicing was assessed as previously reported.^17^ Briefly, RNA was extracted, followed by cDNA synthesis as described above. *Xbp1* transcripts were amplified with the following primers: Forward, *ACACGCTTGGGAATGGACAC*; Reverse, *CCATGGGAAGATGTTCTGGG*. Then PCR products were resolved on a 2% agarose gel. The size of unspliced form (*Xbp1-u*) is 171 bp and spliced one (*Xbp1-s*) is 145 bp.

### RNA sequencing

RNA from freshly isolated intestinal crypts (*n* = 4 for each genotype) was converted into cDNA libraries using the Ovation^®^ RNA-Seq System V2 kit (NuGEN). High-throughput sequencing was performed using Illumina HiSeq X 10 for 4 biological replicates, respectively. For each sample, the RNA-seq data were mapped to mm10 genome by TopHat v1.4.1 with no more than 2 mismatches, and then only the uniquely mapped reads were used to estimate the expression values in gene level by RPKM. Statistically significant test of differentially expressed genes was performed by DEseq with R. Genes with absolute log2-transformed fold changes greater than 1.7 were regarded as differentially expressed genes and a threshold of *p* value < 0.01 was used. Hierarchical clustering of log2-transformed RPKMs was generated by Cluster 3.0 and visualized by Java TreeView.

### Gene set enrichment analysis (GSEA)

GSEA was done using GSEA software (Broad Institute of MIT and Harvard). Genes were ranked according to their log2 (Fold change) values and analyzed using the “pre-ranked” mode of the GSEA software using the following parameters: -mode Max_probe -norm meandiv -nperm 1000 -scoring_scheme weighted -set_max 500 -set_min 15.

### 16S rRNA gene sequencing and analysis

Cecal luminal contents (*n* = 6 for each genotype) and ileal luminal contents (*n* = 2 for each genotype) were collected, and genomic DNA was extracted and purified by using QIAamp DNA Stool Mini Kit (QIAGEN) following manufacture’s instruction. The V3–V4 region of the bacterial 16S rRNA gene was amplified with barcode-indexed primers (338F and 806R). PCR products were purified and sequenced using the Illumina HiSeq platform with paired-end reads (Berry Genomics). Taxonomic classification of OTUs was made using the QIIME software suite and the related 16S database SILVA, and the relative abundance of every taxon was determined. Statistical differences between groups were calculated using PERMANOVA and 999 permutations.

For comparison of microbiota burdens after antibiotics treatment, bacterial genomic DNA was extracted as described above and quantified using a Nanodrop spectrophotometer (Thermo Fisher). SYBR green (Bimake) qPCR for bacterial 16S rRNA genes was performed using primers 515F and 806R. Reactions were performed and monitored using a CFX96 platform (Bio-Rad). Absolute bacterial 16S copy number was quantified using standard curves generated from qPCR of whole 16S gene amplicons purified from *E. coli*.

### Quantification and statistical analysis

Comparisons of villi length, colon length, and the number of epithelial cells in the intestine were performed in a double blinded manner. A minimum of 3 pictures was analyzed per animal. Statistical analyses were performed using GraphPad Prism software (Graphpad, La Jolla, CA). Two-tailed unpaired Student’s *t*-tests and a one-way ANOVA were used for comparison between two groups and three or more groups, respectively. Wilcoxon’s rank sum test was used for comparison of body weight. *P* values < 0.05 were considered statistically significant. Bar graphs represent mean ± SEM. All sample numbers (*n*) listed in legends represent biological replicates.

## Supplementary information

Supplementary information

## Data Availability

Raw NGS data were deposited to the NCBI SRA database. The data will be released upon publication. All other data of this study are available from the corresponding authors upon reasonable request.
