# Supplementary material for: Emc3 maintains intestinal homeostasis by preserving secretory lineages
Source: Mucosal Immunol. 2021 Mar 30;14(4):873–86. doi: 10.1038/s41385-021-00399-2 (PMC8222001; doi:10.1038/s41385-021-00399-2)
Supplement: Supplementary file 1 — Supplementary information [file 41385_2021_399_MOESM1_ESM.pdf]

Supplementary Information

Emc3 maintains intestinal homeostasis by preserving secretory lineages

Huang et al.

Supplementary Figure 1-5

Supplementary Table 1

Supplementary Figure 1

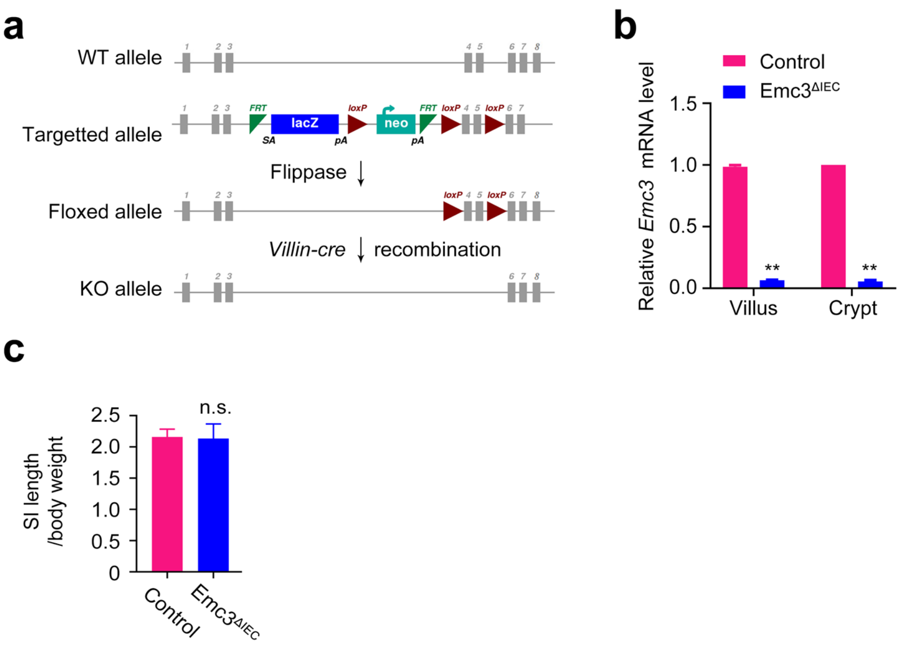

Supplementary Figure 1 | Generation of Emc3 conditional knockout mice.

(a) Experimental scheme of gene targeting strategy for Emc3.

(b) Expression of Emc3 in villus and crypt examined by qPCR. n=3 for each genotype. Statistical data represent mean ± SEM. Student's t-test: $^{**}p < 0.01$.

(c) Length of small intestine (cm) versus body weight (g). n=3 for each genotype. Statistical data represent mean ± SEM. Student's t-test: n.s. not significant.

Supplementary Figure 2

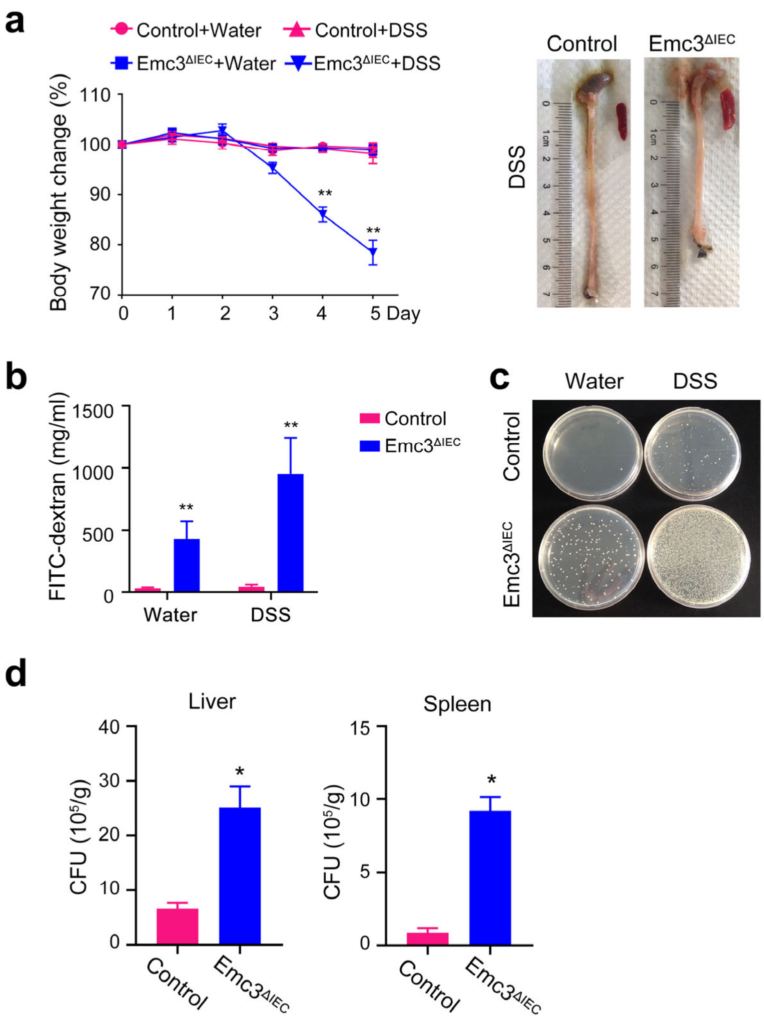

Supplementary Figure 2 | Emc3 is required to protect mice from colitis.

(a) Body weight change and representative images of DSS treated colon. n=7 for each group. Wilcoxon's rank sum test: **p < 0.01.

(b-c) Epithelium permeability shown by serum levels of FITC-dextran **(b)** and bacterial colonies in mesenteric lymph nodes **(c)**. n=3 for each group. Statistical data represent mean ± SEM. Student's t-test: **p < 0.01.

(d) Counting of cultured bacteria in liver and spleen lysate 48 hr after S. Tm infection. n=3 for each genotype. Statistical data represent mean ± SEM. Student's t-test: *p<0.05.

Supplementary Figure 3

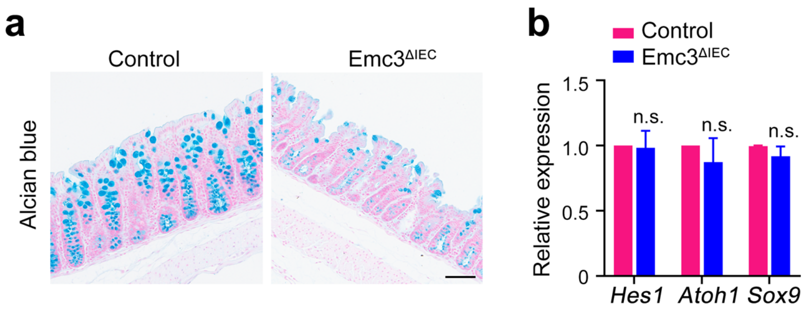

Supplementary Figure 3 | Depletion of goblet cells in *Emc3*$^{\Delta IEC}$ mice.

(a) Representative images of AB-stained goblet cells in colonic sections. Scale bar, 50 μm.

(b) Relative expression of enterocyte progenitor cell marker (*Hes1*) and secretory progenitor cell marker (*Atoh1*) and Paneth regulator (*Sox9*). n=3 for each genotype. Statistical data represent mean ± SEM. Student's t-test: n.s. not significant.

Supplementary Figure 4

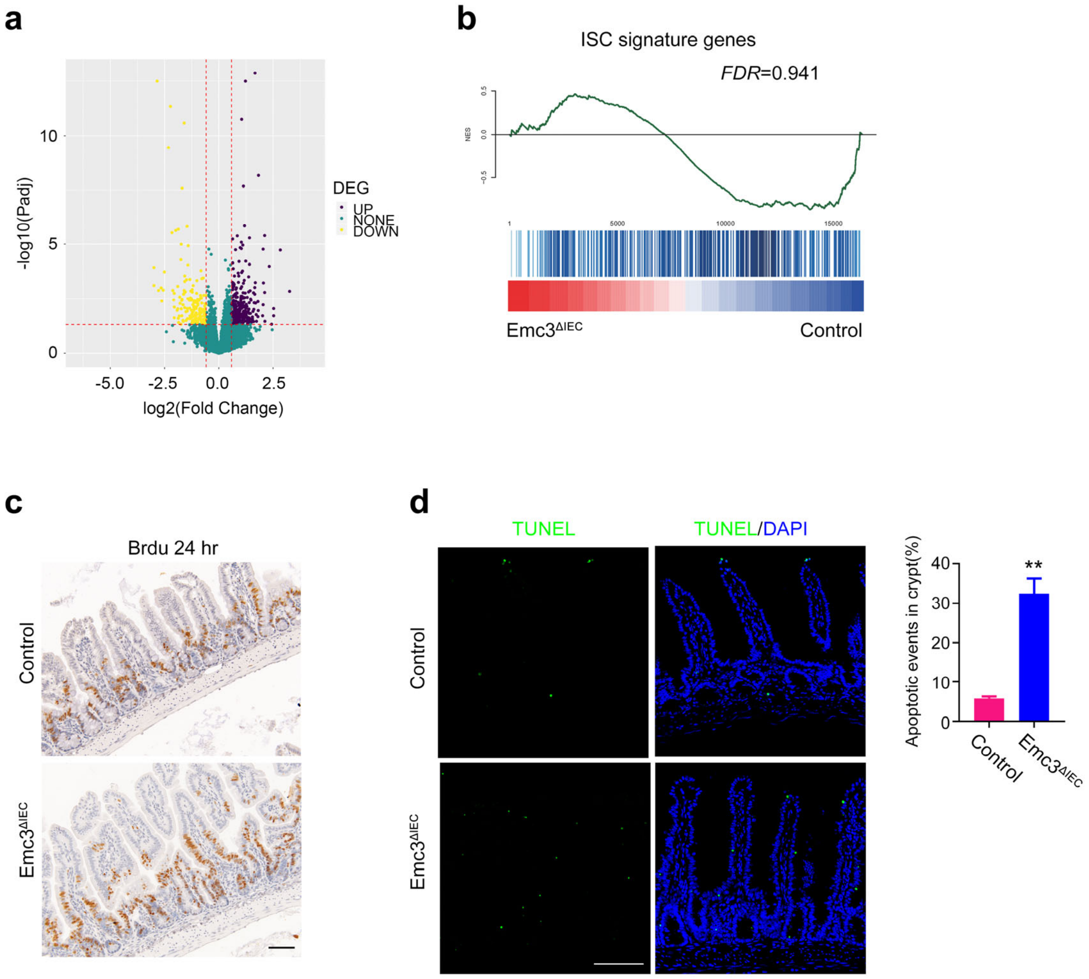

Supplementary Figure 4 | Intestinal stem cells are not impaired in *Emc3*$^{\Delta IEC}$ mice.

(a) Volcano plot displaying the averaged log2 fold change for RNA-seq data of control and *Emc3*$^{\Delta IEC}$ crypts.

(b) GSEA of ISC signature genes in *Emc3*$^{\Delta IEC}$ versus control crypts.

(c) BrdU incorporation (24 hr after injection) assay. Scale bar, 50 μm.

(d) Apoptosis in ileal sections determined by TUNEL analysis. n=3 for each genotype. Statistical data represent mean ± SEM. Student's t-test: **p < 0.01. Scale bar, 50 μm.

Supplementary Figure 5

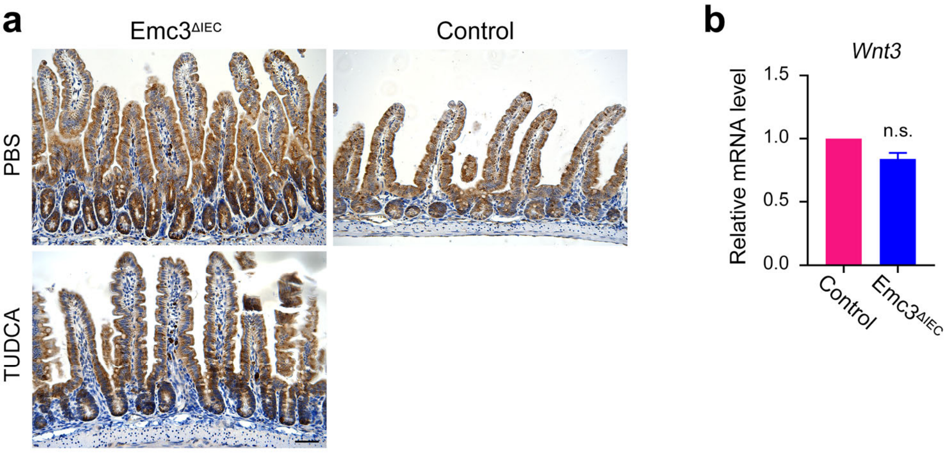

Supplementary Figure 5 | TUDCA alleviates ER stress in *Emc3^{ΔIEC}* mice.

(a) Immunostaining for Bip protein. Scale bar, 50 µm.

(b) Quantification of *Wnt3* expression from TUDCA administered mice. n=3 for each genotype. Statistical data represent mean ± SEM. Student's t-test: n.s. not significant.

Supplementary Figure 6

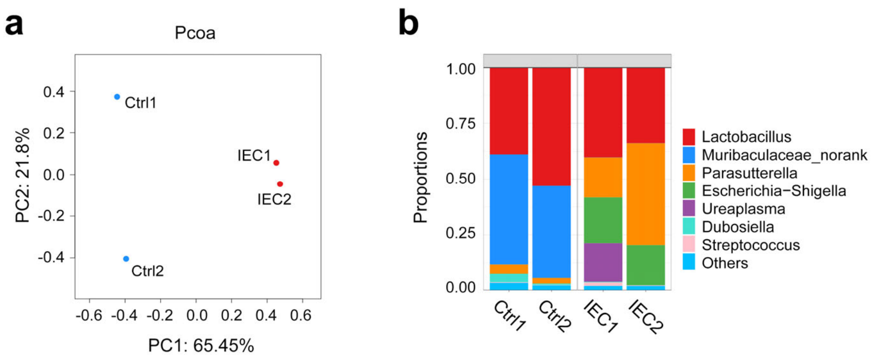

Supplementary Figure 6 | Alteration of microbiota composition in *Emc3*$^{\Delta IEC}$.

(a) PCoA analysis of ileal luminal microbiota from separately housed adult *Emc3*$^{\Delta IEC}$ and control littermates. n=2 for each genotype.

(b) Composition of ileal luminal microbiota at genus level.

Supplementary Table 1 | Primers for RT-qPCR.

Emc3	GTCCTGCCCATCGTTATCAT	ATTTTCCCTGAGGACTCTGC
Atoh1	GAGTGGGCTGAGGTAAAAGAGT	GGTCGGTGCTATCCAGGAG
Gfi1	AGAAGGCGCACAGCTATCAC	GGCTCCATTTTCGACTCGC
Klf4	GTGCCCCGACTAACCGTTG	GTCGTTGAACTCCTCGGTCT
Agr2	GGAGCCAAAAAGGACCCAAAG	CTGTTGCTTGTCTTGGATCTGT
Spink4	TGCCTGACCCGGATGAAAAC	ATGGCTTGAGTGCACCTCTG
Pdia5	GACCCGCAATAACGTGCTG	CTCGGTCATACTGCATGTGAAA
Zg16	CTCGGCCTCTGCTAATTCCAT	GCACCTGGAGACCTACTATGT
Muc2	GTCCGAAGTGTTACCCTGGA	CCAGGAGTGGAGAAGGTCAG
TFF3	TTGCTGGGTCCTCTGGGATAG	TACACTGCTCCGATGTGACAG
Spdef	GGACGGACGACTCTTCTGACAG	GCTCCTGATGCTGCCTTCTCC
Clca1	CTGTCTTCCTCTTGATCCTCCA	CGTGGTCTATGGCGATGACG
Lact	CGTCTGCTTCCTATCAGGTTGAA	GTGGGAAAATGTGTCCCAGATACT
Apli	AACTCACCTCATGGGCCTCTT	GGGTTTCGGTTGGCATCATA
Sis	GCTATCGCTCTTGTTGTGGTT	TTCCAGGACTAGGGGTTGAAG
Chr-A	ATCCTCTCTATCCTGCGACAC	GGGCTCTGGTTCTCAAACACT
Dclk1	TCCACCGGAATTGAACTCGG	GGGAGCGAACAGTCTCAGA
Trpm5	CCAGCATAAGCGACAACATCT	GAGCATACAGTAGTTGGCCTG
Lyz1	GAGACCGAAGCACCGACTATG	CGGTTTTGACATTGTGTTCGC
MMP7	CTGCCACTGTCCCAGGAAG	GGGAGAGTTTTCCAGTCATGG
Cryptdin1	AAGAGACTAAAACTGAGGAGCAGC	CGACAGCAGAGCGTGTA
Cryptdin5	AGGCTGATCCTATCCACAAAACAG	TGAAGAGCAGACCCTTCTTGGC
Defa24	CAAGAGGCTGCAAAGGAAGAGAAC	TGGTCTCCATGTTCAGCGACAGC
Wnt3	CTCGCTGGCTACCCAATTTG	CTTCACACCTTCTGCTACGCT
Wnt11	GCTGGCACTGTCCAAGACTC	CTCCCGTGTACCTCTCTCCA
EGF	AGCATCTCTCGGATTGACCCA	CCTGTCCCGTTAAGGAAAACTCT
Dll4	TTCCAGGCAACCTTCTCCGA	ACTGCCGCTATTCTTGTCCC
Dll1	CAGGACCTTCTTTCGCGTATG	AAGGGGAATCGGATGGGGTT
Lgr5	CGGGACCTTGAAGATTTCCT	GATTCGGATCAGCCAGCTAC
Olfm4	CGAGACTATCGGATTCGCTATG	TTGTAGGCAGCCAGAGGGAG
Ascl2	TGCCGCACCAGAACTCGTAG	ACTCCAGACGAGGTGGGCAT
Hes1	CCAGCCAGTGTCAACACGA	AATGCCGGGAGCTATCTTTCT
Sox9	GCCAGATGGACCCACCAGTAT	TCCAAACAGGCAGGGAGATTC
Gapdh	CATGGCCTTCCGTGTTCCTA	CCTGCTTCACCACCTTCTTGAT
